# Acetylation and Phosphorylation in the Regulation of Hypoxia-Inducible Factor Activities: Additional Options to Modulate Adaptations to Changes in Oxygen Levels

**DOI:** 10.3390/life14010020

**Published:** 2023-12-21

**Authors:** Martina Minisini, Emanuele Cricchi, Claudio Brancolini

**Affiliations:** Lab of Epigenomics, Department of Medicine, Università degli Studi di Udine, 33100 Udine, Italy; martina.minisini@uniud.it (M.M.); cricchi.emanuele@spes.uniud.it (E.C.)

**Keywords:** hypoxia, phosphorylation, HIF-1 alpha, HIF-2 alpha, HIF-3 alpha, acetylation, HDACs, KATs

## Abstract

O_2_ is essential for the life of eukaryotic cells. The ability to sense oxygen availability and initiate a response to adapt the cell to changes in O_2_ levels is a fundamental achievement of evolution. The key switch for adaptation consists of the transcription factors HIF1A, HIF2A and HIF3A. Their levels are tightly controlled by O_2_ through the involvement of the oxygen-dependent prolyl hydroxylase domain-containing enzymes (PHDs/EGNLs), the von Hippel–Lindau tumour suppressor protein (pVHL) and the ubiquitin–proteasome system. Furthermore, HIF1A and HIF2A are also under the control of additional post-translational modifications (PTMs) that positively or negatively regulate the activities of these transcription factors. This review focuses mainly on two PTMs of HIF1A and HIF2A: phosphorylation and acetylation.

## 1. Introduction

### The Hypoxia Response

Human cells need O_2_ to regenerate ATP, to multiply, and to survive. When O_2_ availability decreases, complex adaptive responses are initiated that conserve oxygen consumption by reducing oxidative phosphorylation in the mitochondria, and support glycolysis. Angiogenesis is stimulated and cell proliferation is reduced. The transcription factors HIF1A/HIF-1a and EPAS1/HIF2A/HIF-2a are key players in of the adaptive response to O_2_ depletion [1,2,3,4].

**Figure 1:**
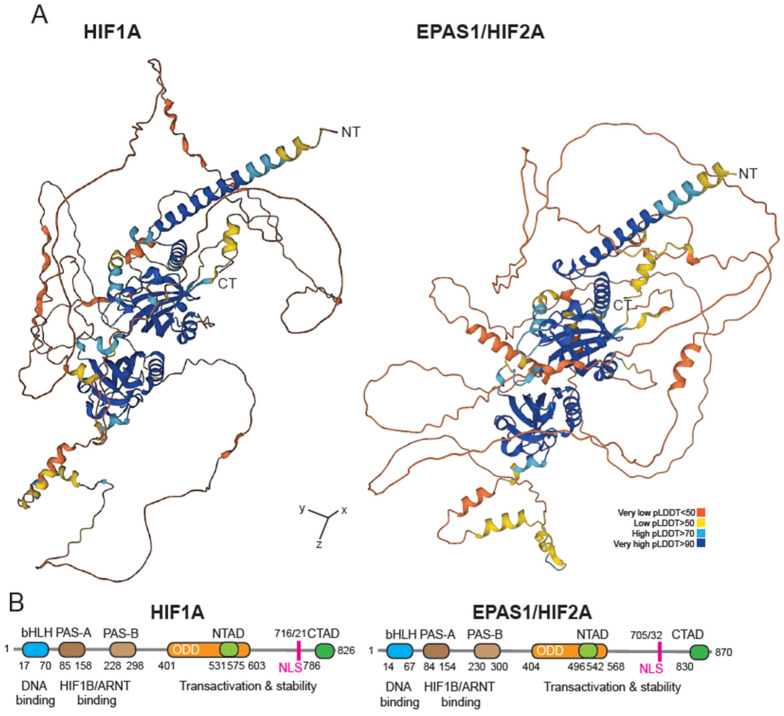
HIF1A and HIF2A/EPAS1 proteins. (**A**) AlphaFold prediction of HIF1A and HIF2A/EPAS1. The colours indicate the different per-residue confidence score (pLDDT) as indicated. Some regions below 50 pLDDT may be unstructured in the isolation. https://alphafold.ebi.ac.uk. (**B**) Schematic representation of HIF1A and HIF2A/EPAS1. Both proteins contain basic helix–loop–helix (bHLH) and Par-Arnt-SIM (PAS) transcription factor domains that facilitate the heterodimerization with ARNT. They also have N- and C-terminal transactivation domains (N/C-TAD), an oxygen-dependent degradation domain (ODD), and nuclear localization sequence (NLS). Their sequences show a high degree of similarity; amino-terminal (NT) and carboxy terminal (CT).

HIF-1 is a heterodimer composed of the O_2_-sensitive subunit HIF1A and a constitutively expressed subunit HIF1B/HIF-1β, also known as the aryl hydrocarbon receptor nuclear translocator (ARNT) [5]. In selected vertebrate cell types or in cancer cells, another O_2_-sensitive subunit, EPAS1/HIF2A is also expressed (Figure 1). The two subunits share many transcription targets, but there are also genes that are subject to exclusive regulation. In particular, HIF1A and EPAS1/HIF2A can have opposing effects in cancer cells by controlling the transcription of different target genes [6,7].

HIF-3α/HIF3A is the third member of the O_2_-inducible HIF-TFs. Similar to EPAS1/HIF2A, it is specifically expressed in certain tissues [8]. Structurally, HIF3A differs from HIF1A and EPAS1/HIF2A mainly in the carboxy term, where a leucine zipper domain (LZIP) is present, which is involved in protein–protein interactions, while the TAD is absent [9,10]. Ten different HIF3A splice variants are known (HIF-3α1-10), which originate from alternative initiation transcription sites. HIF3A can act as an inhibitor of transcription mediated by HIF1A and EPAS1/HIF2A by competing for binding to HIF1B [10,11].

**Figure 2:**
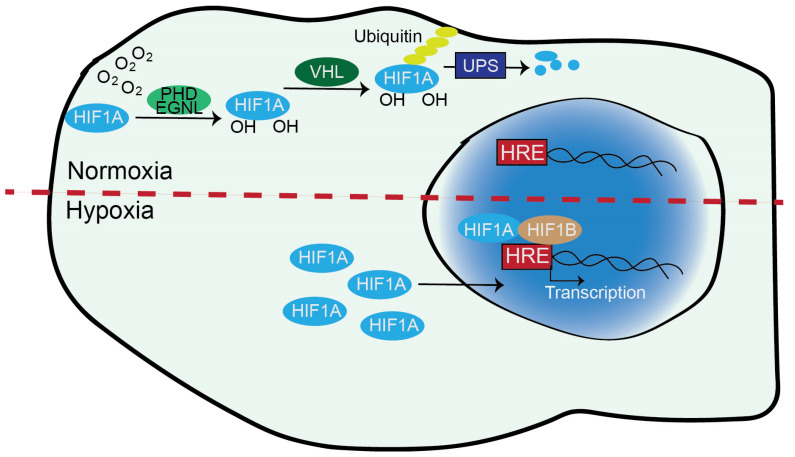
The cellular response to hypoxia. Under normoxia, key prolines of HIF1A are hydroxylated by PHD/EGLN proteins using O_2_ and α-oxoglutarate (also known as α-ketoglutarate). When HIF1A is hydroxylated, it is recognized by VHL, polyubiquitylated, and directed to proteasomal-mediated degradation. Under hypoxia, HIF1A accumulates and associates with ARNT/HIF1B. The heterodimer binds to the HRE consensus motif and supervises the transcription of genes of the adaptive response to low oxygen levels.

O_2_ controls the stability of HIF1A and EPAS1/HIF2A through the action of 2-oxoglutarate (2-OG)-dependent dioxygenases (2-OGDDs) and the prolyl hydroxylases PHD1, PDH2 and PDH3, also known as EGLN2, EGLN1 and EGLN3, respectively (Figure 2). These enzymes hydroxylate key prolines of HIF1A (Pro-402 and Pro-564) and of EPAS1/HIF2A (Pro-405 and Pro-531) using O_2_ and α-oxoglutarate [1,12,13]. When HIF1A and EPAS1/HIF2A are hydroxylated, they are recognized by VHL, a tumour suppressor gene responsible for von Hippel–Lindau disease. VHL is the substrate recognition subunit of an ubiquitin E3-ligase complex that directs HIF1A and EPAS1/HIF2A to proteasomal degradation [4]. This multiprotein complex also includes Cullin-2 (Cul-2), Elongin-1, Elongin-2 and Ring-Box 1 (RBX1). It polyubiquitylates HIF1A at K532, K538, K567 or EPAS1/HIF2A at residues K497, K503, K512 [14].

When the O_2_ content decreases, hydroxylation cannot take place, and HIF1A/EPAS1/HIF2A rapidly accumulate. After dimerization, the mature TFs migrate into the nucleus and bind to E-box-like hypoxia response elements (HREs) in the promoter region of hundreds of target genes (Figure 2). These genes represent the hypoxia response, and HIFs support their transcription and adaptation to the altered environment [15]. Control by the ubiquitin–proteasome system (UPS) is a key mechanism for cellular adaptation to varying O_2_ concentrations.

## 2. HIF1A and HIF2A: Not Only Protein Hydroxylation

Regulating the hydroxylation and stability of HIF1A and EPAS1/HIF2A in response to O_2_ is a rapid and efficient strategy to link changes in environmental conditions to cellular adaptations. However, other PTMs are also used to modulate HIF1A and EPAS1/HIF2A activities and hypoxia response, mainly by controlling protein stability or enhancing transcriptional activity.

## 3. Phosphorylation

**Figure 3:**
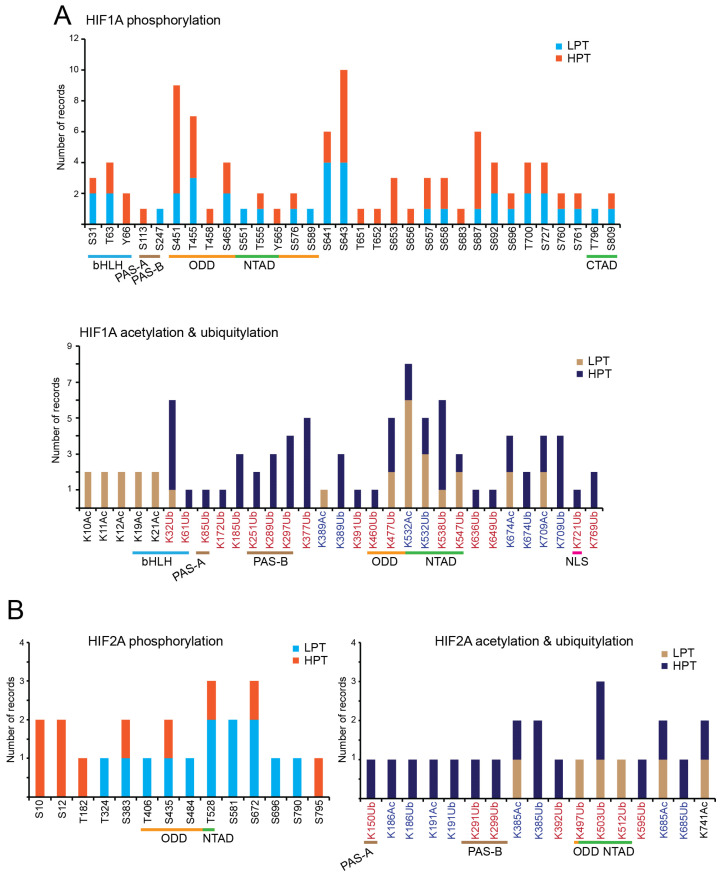
Phosphorylated, acetylated, and ubiquitylated sites in human HIF1A and EPAS1/HIF2A. (**A**) Phosphorylated, acetylated, and ubiquitylated sites in human HIF1A, determined by PhosphoSitePlus https://www.phosphosite.org/proteinAction.action?id=4987&showAllSites=true (accessed on 14 November 2023). HTP: Number of records in which the specific PTM was only detected by mass spectrometry. LPT: number of records where the specific PTM was experimental demonstrated without mass spectrometry [20]. The different domains are indicated. (**B**) Phosphorylated, acetylated and ubiquitylated sites in human EPAS1/HIF2A, determined by PhosphoSitePlus https://www.phosphosite.org/proteinAction.action?id=4986&showAllSites=true (accessed on 14 November 2023). HTP: number of records in which the specific PTM was only detected by mass spectrometry. LPT: number of records where the phosphorylation was experimental demonstrated without mass spectrometry [20]. The different domains are indicated.

The regulation of kinase activities in response to hypoxia and the control of phosphorylation of HIFs-TFs in normoxia or hypoxia have been intensively studied. Several kinases have been reported to have HIF1A and EPAS1/HIF2A as substrates. This implies that alternative signalling pathways can be used to modulate the activity of these TFs. Several residues have been reported as targets of phosphorylation (Figure 3A,B) [16]. The possibility that phosphorylation could affect the synthesis, stability, and activity of HIF1A was observed in early studies with various inhibitors, including tyrosine phosphatase inhibitors [17]. Several studies have subsequently confirmed that receptor tyrosine kinase signalling or cytoplasmic/nuclear tyrosine kinases can indirectly influence HIF1A [18,19]. However, there is no experimental evidence that tyrosine kinases can directly phosphorylate HIF1A.

### 3.1. GSK3b: Another Route to Degradation

The serine-threonine kinase GSK3b can phosphorylate HIF1A at multiple residues within the oxygen-dependent degradation (ODD) domain. This leads to VHL independent polyubiquitylation and degradation of HIF1A [21,22,23]. The ubiquitin ligase FBW7-E3 is involved in the GSK3b-dependent degradation of HIF1A in both normoxia and hypoxia. Deubiquitylases are often part of E3–ligase–ligase multiprotein complexes which fine-tune the degradation option [24]. In the case of HIF1A, the deubiquitylase USP28 can antagonize the FBW7-E3 ligase to prevent HIF1A from degradation [14,23,25].

### 3.2. Cell Cycle Kinases

Hypoxia reduces cell proliferation in a variety of cell types, including cancer cells. HIF1A can directly control cell cycle progression [2]. It is therefore not surprising that various kinases that are active during the cell cycle can phosphorylate HIF1A. Polo-like kinase 3 (PLK3), a family of serine-threonine kinases that contribute to the control of mitosis but also have additional non-mitotic functions, regulates the stability of HIF1A in normoxia [26]. Serine 576, within the ODD domain, and serine 657, adjacent to the nuclear export sequence (NES), are the sites that are phosphorylated by PLK3 [27]. By phosphorylating these sites, PLK3 exerts a negative influence on the stability and activity of HIF1A [28].

During the cell cycle under normoxia, HIF1A is also a target of regulation by the CDKs (cyclin-dependent kinases), the most important regulators of cell cycle progression. Opposite effects on the stability of HIF1A have been described for CDK1 and CDK2 [29]. According to one study, the lysosomal degradation pathway is involved in the control of HIF1A levels. More specifically, CDK2 favours the lysosomal degradation of HIF1A, while CDK1 hinders it [29]. However, another group of researchers confirmed that CDK1-dependent phosphorylation of HIF1A can underpin its activity, albeit by suppressing proteasome-mediated degradation [30]. Finally, it should be mentioned that CDK2 can promote the activity of HIF1A activity in certain cancer cells [28]. It can be concluded that normal cell cycle progression cycle requires tight regulation of HIF1A levels.

In endothelial cells, CDK5, which is not involved in cell cycle control, can phosphorylate HIF1A at serine 687 and thus stabilize it [31].

### 3.3. PKA

A role of PKA in the regulation of HIF1A phosphorylation was first reported in endothelial cells exposed to intermittent hypoxia [32]. Subsequently, PKA was shown to phosphorylate T63 and S692 on HIF1A, inhibiting its proteasome-mediated degradation and promoting its transcriptional activation. PKA also stimulates the binding of KAT (lysine acetyl-transferase) p300/KAT3B to the carboxy terminal TAD domain of HIF1A to sustain transcriptional activation [33]. In this way, phosphorylation can coordinate acetylation.

### 3.4. HIF1A Phosphorylation and the DNA Damage Response (DDR)

In proliferating cells, hypoxia can also engage elements of the DNA damage response (DDR) by inducing replication stress [34,35,36]. Members of the phosphoinositide 3-kinase-related kinase (PIKKs) family DNA-PK, ataxia telangiectasia-mutated (ATM), and ataxia-telangiectasia and Rad3-related kinase (ATR) can modulate HIF1A levels and activities [33,34,35]. ATM has been shown to phosphorylate HIF1A at S696, a process associated with downregulation of mTORC1 signalling [34]. Instead, ATR is required for efficient translation of *HIF1A* mRNA, via an as yet undetermined mechanism [35,36].

An alternative indirect mechanism has been proposed to explain the link between hypoxia, DDR and HIF1A. The histone variant H2AX interacts with HIF1A and stabilizes it, protecting it from nuclear export and degradation. Monoubiquitylation and phosphorylation of H2AX, which are strictly mediated by hypoxia-induced E3 ligase activity of TRAF6 and ATM, can activate HIF1A signalling and promote tumorigenesis [37]. Another indirect mechanism utilized by ATM/ATR to maintain HIF1A involves seryl-tRNA synthetase (SerRS). SerRS can regulate blood vessel formation by repressing the transcription of VEGFA, independent of its aminoacylation activity. The proposed mechanism is that when SerRS is not phosphorylated by ATM/ATR, it can compete with HIF1A for binding to DNA, and switch off the hypoxic genetic program [38].

### 3.5. Other Kinases

ERK1/2 can also phosphorylate both HIF1A and EPAS1/HIF2A. Phosphorylation enhances transcriptional activity by inhibiting nuclear export. In fact, the phosphorylated residues (Ser641/643 in HIF1A and S672 in EPAS1/HIF2A) are located near a non-canonical NES and affect binding to the exportin CRM1 [39,40].

Casein kinase 1 (CK1) has also been described as an upstream regulator of HIF1A activities. Casein kinase 1δ (CK1δ) controls S247 phosphorylation within the N-terminal heterodimerization domain of HIF1A. This results in impaired formation of the HIF1A/HIF1B complex and reduced response to hypoxia [41]. CK1δ is also involved in the phosphorylation of EPAS1/HIF2A, but unlike HIF1A, this phosphorylation enhances transcriptional activity. S383 and T528 are the residues phosphorylated in vitro. As with ERK1/2, the proposed mechanism involves the regulation of CRM1-dependent nuclear export under hypoxia [42].

The PIM (Proviral Integration site for Moloney murine leukemia virus) family of serine threonine kinase are pro-oncogenic factors that control cell cycle progression, proliferation, and survival [43]. They also promote tumour angiogenesis by controlling the phosphorylation of HIF1A and EPAS1/HIF2A. HIF1A is phosphorylated at threonine 455 and HIF2A at serine 435. In both cases, protein stability is increased, even under normoxic conditions. Phosphorylation impairs the binding of prolyl hydroxylases and the canonical pathway of proteasomal-mediated degradation [44].

Using LC/MS/MS-based analysis, Ser451 was identified within the ODD of HIF1A as a target of phosphorylation under hypoxic conditions. This phosphorylation is important for the maintenance of HIF1A levels by inhibiting its interaction with PHD and pVHL. In this way, tumour growth is supported [45]. The protection of HIF1A degradation may depend on the binding of the chaperone HSP90, but the kinase involved is unknown.

Finally, it was recently reported that the PKB/AKT kinase phosphorylates HIF3A1 directly at serine 524 in the ODD domain to regulate its stability. Mutagenesis at this site impairs cell proliferation and survival, leading to defects in proliferation, colony formation and cell adherence [46].

## 4. Acetylation

Lysine acetylation is a widespread and conserved PTM that regulates virtually all cellular processes, from bacteria to human cells [47]. Although much attention has been paid to the acetylation of histones in the context of chromatin organization and epigenetics, it is now clear that hundreds of different proteins can be acetylated in different contexts and cellular compartments [48].

This PTM is reversible and is antagonistically controlled by two families of enzymes [49,50]. The KATs (lysine acetyl transferases) catalyse the transfer of an acetyl group from acetyl-CoA to the e-amino group of certain lysine residues. Their action is counteracted by KDACs (lysine deacetylases), which are mostly known as HDACs (histone deacetylases).

KATs can be divided into three main families based on their homology to yeast orthologs and their catalytic mechanism. The three families are (i) the p300/CREB-binding proteins (p300/CBP); (ii) the GCN5-related N-acetyltransferases (GNAT); and (iii) the MOZ, Ybf2, Sas2 and Tip60 (MYST) family [51,52]. In addition, several protein complexes have been reported to possess lysine acetyltransferase activity. They are referred to as non-canonical KATs [49].

Similarly, the 18 human KDACs/HDACs can be divided into 5 different subfamilies based on their homology to yeast orthologs and their catalysis mechanism. Class I includes HDAC1, HDAC2, HDAC3 and HDAC8, which share homology with Rpd3. Class IIa includes HDAC4, HDAC5, HDAC7 and HDAC9. Class IIb includes HDAC6 and HDAC10. Class IIa and class IIb have a common homology with Hda1. Class III groups, Sirt1, 2, 3, 4, 5, 6 and 7, also known as silent information regulators (SIR), are homologous to Sir2 in yeast. Class IV contains only HDAC11, as it has sequence similarities with the family members of classes I and II. Classes I, II and IV members are zinc-dependent enzymes, while class III members are NAD^+^-dependent [50,53,54].

Class IIa HDACs in vertebrates are characterized by a Tyr/His substitution in the catalytic site, which drastically reduces (or almost eliminates) the catalytic activity against acetyl-lysine that characterizes class IIa HDACs in vertebrates. However, by binding to class I HDACs via the deacetylase domain, and in particular to the NCOR1-NCOR2-HDAC3 complex, they can monitor lysine deacetylation [55,56].

### 4.1. HIF1A and EPAS1/HIF2A Acetylation

Various residues of HIF1A and EPAS1/HIF2A have been experimentally described as acetylation sites or identified by mass spectrometry analysis. Figure 3A,B summarize these data as reported by the PhosphoSitePlus database for HIF1A and EPAS1/HIF2A [20]. The same lysine is also frequently ubiquitylated, suggesting a possible link to the regulation of protein stability. In general, the presence of lysine residues targeted for both acetylation and ubiquitylation is commonly observed in different proteins. This is an evolutionarily conserved competition for a more sophisticated control of protein stability [57,58,59]. In principle, the acetylation of lysines that are signals for the proteasome via K48-linked poly-ubiquitylation, can be considered a factor in protein stabilization factor. This is the case for acetylation at K709 of HIF1A, which increases protein stability by reducing poly-ubiquitylation under both normoxia and hypoxia conditions. This acetylation is mediated by p300/KAT3B and antagonized by HDAC1 but not HDAC3 [60]. However, the consequences of lysine acetylation could be different depending on which residues are modified, especially if the region is under the control of some E3 ligases.

KATs and HDACs may act upstream of HIF1A and EPAS1/HIF2A to control the acetylation status of selected lysines. In addition, KATs and HDACs can also be partners and downstream effectors of these TFs to locally modify chromatin (histone acetylation) to promote or repress gene transcription [61]. In this review, KAT and KDACs are discussed as upstream regulators of HIF1A and EPAS1/HIF2A.

The p300/CBP-associated factor (PCAF/KAT2B) was originally identified as part of a multiprotein complex with HIF1A that controls the transcription of hypoxia-responsive genes [62]. PCAF/KAT2B acetylates HIF1A at K532 and possibly other sites to regulate its stability, and selectively directs HIF1A to a subset of target genes [63].

EPAS1/HIF2A is similarly regulated by acetylation. The CREB-binding protein (CREBBP/CBP/KAT3A) can interact via an enzyme/substrate interaction and contributes to HIF2A-mediated EPO transcription. Acetylation mediated by KAT3A is reversed by SIRT1, and this cycle enhances (in an undetermined manner) HIF2A activity [64].

A general increase in HIF1A acetylation was observed in response to DNA damage and LPS treatment, which correlates with increased protein stability in macrophages. The KAT Tip60/KAT5 was involved in the regulation of acetylation and protein stability in response to LPS. Tip60 binds to HIF1A, and together with the CDK8 mediator complex is required for efficient expression of most genes under HIF1A control during hypoxia [65].

In a comparative study, K674 of HIF1A and K741 of HIF2A were found to be acetylated by PCAF/KAT2B and CBP/KAT3A, respectively. These residues are deacetylated by SIRT1, leading to a reduction in the transactivation activity of both TFs, but in the case of EPAS1/HIF2A depending on the cell line [66].

In general, several studies show a positive effect of lysine acetylation on the stability and transcriptional activity of HIF1A and EPAS1/HIF2A. As a logical consequence, one could argue that HDACs/KDACs should act as negative regulators of HIF1A and EPAS1/HIF2A. However, the available literature suggests a more complex scenario.

### 4.2. HDACs and Hypoxia

The involvement of HDACs in the regulation of the cellular response to hypoxia soon attracted interest. Initial studies suggested that HDAC1 exerts a pro-hypoxia function by regulating the expression of TP53 and von Hippel–Lindau [67]. Non-selective HDAC inhibitors (HDACIs) impeded the hypoxia response and the neovascularization process [67,68]. However, since HDACs can act as both regulators and effectors, it soon became clear that HDACIs act at multiple levels [69,70,71]. A negative influence of HDACs on HIF1A-dependent transcription was demonstrated in a study with the viral oncoprotein E7 of human papillomaviruses (HPV). E7 was able to displace the binding of class I and class IIa HDACs to HIF1A, while it did not affect the binding of p300/KAT3B and of PCAF/KAT2B [72]. However, opposite results have also been reported with a positive effect of HDACs on HIF1A activities under hypoxia (see below).

HDACs can also be a partner of HIF1A to modulate a repressive genetic program [73,74]. Moreover, it is important to remember that HDACs can also act independently of HIF1A during hypoxia. For example, in the regulation of transcription elongation, a key process that determines efficient transcription and gene expression, phosphorylation of the carboxy terminus of RNA polymerase II (RNAPII) by transcription elongation factor b (P-TEFb) is important to promote productive elongation and gene transcription. Active PTEFb is composed of the serine-threonine kinase CDK9 and cyclin T1 or cyclin T2, two distinct regulatory subunits. P-TEFb can be kept inactive when associated in a complex containing the small nuclear RNA 7SK and the HEXIM1 protein [75]. Hypoxia has been reported to inhibit the elongation of transcription. Mechanistically, under hypoxia, HDAC3/NcoR complexes colocalize in the nuclei with CDK9 cyclin T1, which is deacetylated. In this way, the formation of the inactive complex of CDK9/cyclin T1 with HEXIM1 is favoured, and the expression of several genes is downregulated [76].

## 5. Class I HDACs

Studies on the involvement of class I HDACs as upstream regulators of HIF1A and EPAS1/HIF2A activities suggest a possible role of HDAC1 and HDAC2 and exclude a contribution of HDAC3. However, it should be considered that HDAC3 may contribute to the deacetylase activities of class IIa HDACs, as discussed in Section 6.

HDAC1 and HDAC2 are often partners in various multiprotein complexes containing scaffolding factors that are required both to enhance their enzymatic activity and to mediate interaction with selected TFs or epigenetic readers. In addition, either HDAC1 or HDAC2 has been reported to act independently as part of different complexes [54].

Hypoxia could affect the activities of HDAC1 and HDAC2 activities via protein kinase CK2-driven phosphorylation, which contributes to the downregulation of pVHL and stabilization of HIF1A [77]. The involvement of HDAC1 in the control of HIF1A acetylation has also been implicated in a complex with metastasis-associated protein 1 (MTA1), a subunit of the nucleosome remodelling and histone deacetylation complex (NuRD) [78].

PTMs can often be coordinated by the activities of multiprotein complexes. Lysine demethylases may be part of the NuRD complex and influence the activity of HIF1A. LSD1 can indirectly regulate the expression of MTA1 through the control of the MYC oncogene, which increases the interaction with HIF1A, and in this way favours the deacetylation of HIF1A. In addition, LSD1 autonomously demethylates HIF1A at K391, a PTM that counteracts its ubiquitylation. Finally, LSD1 can also suppress PHD2-induced HIF1A hydroxylation by reversing Set9-mediated HIF1A methylation [79].

In hepatitis B virus (HBV)-associated hepatocarcinogenesis, the HBV core protein and a regulatory X protein (HBx) enhance the expression of the MTA1 and HDAC1 genes. The MTA1 and HDAC1/2 complex can bind to HIF1A in vivo in the presence of HBx. Deacetylation of the oxygen-dependent degradation domain of HIF1A and dissociation of prolylhydroxylases and the von Hippel–Lindau binding resulted in the stabilization of the protein. Although no data were available on the specific lysine residues that are deacetylated, the stability of the K532R-HIF1A mutant was not affected, even after treatment with HDACIs [80]. Since this lysine may also be subject to ubiquitylation, it is difficult to extrapolate a clear contribution of acetylation to this residue.

As described above, an opposite scenario was observed in the case of K709. Deacetylation by HDAC1 at this residue promotes ubiquitylation and reduces HIF1A activities in both normoxia and hypoxia, leading to a vigorous debate [60,81].

Recently, a role of HDAC8 in the stability of HIF1A has been proposed. Silencing of HDAC8 increases HIF1A protein levels in normoxia as well as in hypoxia or under cobalt chloride (CoCl_2_)-induced hypoxic conditions. A similar effect was induced by the HDAC8 inhibitor PCI-34051. This effect appears to play out at the level of protein stability and is associated with a general increase in HIF1A acetylation [82]. It would be interesting to clarify on which lysines HDAC8 exerts its effect.

## 6. Class IIa HDACs and the Regulation of the Hypoxic Response

Several studies have investigated the contribution of class IIa to the acetylation of HIF1A. However, the relationships between hypoxia and HDACs may be reciprocal. For example, ARNT/HIF1B deficiency leads to decreased HDAC activity, increased global histone acetylation, and altered subcellular localization of class IIa HDACs [83]. In the next sections, the contribution of each member of the class IIa family to HIF1A activity will be discussed.

### 6.1. HDAC4

In the VHL-deficient human renal cell carcinoma cell line UMRC2, HDAC4 and HDAC6 were isolated as part of a complex with HIF1A. Interfering with these HDACs decreased protein expression and transcriptional activity of HIF1A. However, only downregulation of HDAC4 led to increased acetylation of HIF1A, as demonstrated by co-immunoprecipitation experiments. [84]. The interaction between HIF1A and HDAC4 was also confirmed in another study using an in situ proximity ligation assay and fluorescence microscopy [85].

Another study confirmed that HDAC4 can regulate the acetylation and stability of HIF1A. The same authors excluded a contribution of HDAC1 and HDAC3. Based on the different sensitivity to proteolysis demonstrated by LC-MS/MS analyses, the authors speculated that different lysine residues at the amino terminus of HIF1A appear to be regulated by HDAC4 (lysines 10, 11, 12, 19 and 21). In addition, the silencing of HDAC4 affects the hypoxia-induced increase in glycolysis and resistance to docetaxel chemotherapy [86]. Considering the low/absent enzymatic activity of class IIa HDACs in vertebrates and the exclusion of the involvement of HDAC3, it is unclear which multiprotein complex recruited by HDAC4 is involved in this deacetylation reaction.

In another study, the role of HDAC4 in regulating HIF1A abundance was reconfirmed, but possibly in complex with HDAC3-NCOR1-NCOR2. In this case, a reduction in cell proliferation was only observed in a hypoxic environment when HDAC4 was silenced. Whether this effect is related to HIF1A or to other targets of HDAC4 is unclear [87]. 

HDAC4 can accumulate in the nucleus, when it is present in complex with nucleus accumbens-associated protein-1 (NAC1), a nuclear factor of the BTB/POZ gene family. The authors hypothesized that HDAC4 is stabilized in the nucleus. Indeed, previous studies have indeed shown that HDAC4 is subject to regulation by the UPS [88]. Under hypoxia, higher levels of HDAC4 resulted in reduced HIF1A acetylation and inhibition of UPS-mediated degradation. In this context, the NAC1-HDAC4 axis promotes glycolysis in hypoxic tumour cells [89].

Apart from the UPS, the regulation of HDAC4 levels during hypoxia can also be influenced by other factors. A recent study showed that *HDAC4* mRNA is strongly regulated under hypoxia conditions. In pancreatic cancer cell lines, hypoxia leads to a reduction in N6-methyladenosine (m6A) modification in mRNAs due to increased expression of the m6A eraser ALKBH5 [90]. m6A is the most common modification detected in eukaryotic mRNAs, and it is also observed in several other RNA species [91]. This epigenetic modification can affect almost every step of RNA metabolism, including splicing, transport, translation, and stability [92]. By combining MeRIP-seq and RNA-seq obtained from normoxic and hypoxic cells, the authors defined a hypoxia-related m6A modification signature that controls glycolysis and metastasis. Among the most enriched genes was *HDAC4*, whose expression level was increased under hypoxic conditions and partially in an ALKBH5-dependent manner. A reduction in m6A modification increases the half-life of HDAC4 mRNA, which contributes to an increase in HDAC4 protein under hypoxia. Similar to previous studies, the authors show that HDAC4 can control HIF1A levels. Further experiments are required to elucidate the detailed mechanisms, including which lysine residues are specifically involved in this regulation, and whether deacetylation is the key event [90]. However, it is evident that several mechanisms may contribute to HDAC4 levels and activity under hypoxia.

One particular mechanism has been described for the transcription of the coagulation factor VII (FVII) gene in response to hypoxia. In ovarian cancer, HIF2A regulates FVII expression in complex with Sp1, but without ARNT and in an HRE-independent manner. HDAC4 and KAT p300 are also found in this complex. Paradoxically, HDAC4, but not p300, is required for transcriptional activation [93].

Negative influences of HDAC4 on HIF1A activities have also been reported. We have already discussed the contribution of the viral oncoprotein E7 to HIF1A activities by repressing binding to various HDACs, including HDAC4 [72]. Finally, we should always keep in mind that an epigenetic regulator such as HDAC4 can be part of different multiprotein complexes and is able to regulate various adaptive responses [94]. For example, HDAC4 in combination with RUNX2 can repress the transcription of vascular endothelial growth factor (VEGF), which is a major HIF1A target gene and the most potent pro-angiogenic factor [95]. In summary, although there are several lines of evidence for the involvement of HDAC4 in the cellular response to hypoxia, the mechanisms involved remain to be further defined.

### 6.2. HDAC5

Of class IIa, HDAC5 has the greatest homology to HDAC4, and therefore it is not surprising that it contributes to HIF1A activity. In several cell lines (MCF7, HeLa, Hep3B), silencing HDAC5, but not HDAC1, HDAC3 or HDAC6, leads to a decrease in HIF1A stability. HDAC5 in the cytoplasm contributes to the stabilization of HIF1A in response to hypoxia and to its accumulation in the nucleus [96]. To investigate the involvement of the deacetylase domain, the authors generated a double mutant (C698A/H704A) corresponding to the mutant of HDAC4 (C669A/H675A) in the structural zinc-binding domain. It is important to note that this mutant impairs the binding of HDAC4 to the HDAC3/NCOR1 complex [97]. Therefore, the contribution of HDAC3 in the regulation of HIF1A stability cannot be excluded. As a mechanism, the authors propose that the effect on HIF1A stabilization is mediated by the regulation of HSP70 acetylation [96].

Other studies have reported a repressive effect of HDAC5 on HIF1A expression, although the mechanism has not been defined. Cycles of intermittent hypoxia (IH) have been used as a model to study manifestations of obstructive sleep apnoea (OSA). In this example, downregulation of HDAC3 and HDAC5 occurred, and it has been suggested that this downregulation contributes to the stability of HIF1A by increasing lysine acetylation [98].

The same authors also reported that ROS trigger the degradation of HDAC5 during IH by dephosphorylation of S259. The authors hypothesized that degradation of HDAC5 is responsible for the increase in HIF1A levels, its acetylation, and transcriptional activity [99]. Downregulation of HDAC5 and HDAC6 expression during hypoxia was also observed in adipocytes from humans and mice. RNAi-mediated silencing of these two HDACs mimicked some of the effects of hypoxia [100].

### 6.3. HDAC7

The first studies on the possible involvement of class IIa HDACs in the regulation of hypoxia pointed to a role for HDAC7. It was shown that HDAC7 can bind to HIF1A via the carboxy terminus. Under hypoxic conditions, HDAC7 accumulated in the nucleus as a complex with p300/KAT3B and HIF1A, and stimulated the transcriptional activity of HIF1A in an undetermined manner [101]. When comparing the subcellular localization of epitope-tagged HDAC4, HDAC5 and HDAC7, only HDAC7 accumulated in the nucleus under hypoxia [101]. An interaction between HDAC7 and HIF1A was also observed in other cell models and under different stimuli. In macrophages, HIF1A expression is induced by LPS. HDAC7 and HIF1A interact and synergistically to promote LPS-induced transcription of the *End1* gene [102]. Investigating the regulation of the HIF1A-HDAC7 axis in macrophages in response to inflammatory signalling, a recent study has shown that PKM2 may be part of a ternary complex with HIF1A and HDAC7. HDAC7 can control the acetylation/dimerization of PKM2, leading to its stabilization and nuclear translocation [103].

### 6.4. HDAC9

Few studies have investigated the role of HDAC9 in hypoxia [104]. Similar to the other class IIa members of the HDACs family, HDAC9 has been shown to interact with HIF1A, deacetylating it and maintaining its activities [105]. Additional mechanisms have also been proposed. In liver cancer cell lines, HDAC9 is required to drive efficient HIF1A translation in an unspecified manner, which in turn is mediated by the eukaryotic translation initiation factor eIF3GA. This mechanism is also utilized by SAHA [106]. Finally, mRNA levels of HDAC9 can be dramatically upregulated under 48 h of hypoxia in renal cell carcinoma cell lines. In this context, HDAC9 acts as a downstream epigenetic regulator of H3K27ac levels and gene expression [107].

## 7. Class IIb

The enzymes HDAC6 and HDAC10 form class IIb. HDAC6 has attracted much more attention, and more data are available compared to HDAC10. HDAC6 is structurally unique, contains two catalytic domains, and is predominantly localized in the cytoplasm. In fact, several substrates of HDAC6 are non-histone proteins with cytosolic activities, including α-tubulin, heat shock protein (HSP90), cortactin, peroxiredoxin, etc. [108]. Another distinctive feature of HDAC6 is the presence of the zinc finger ubiquitin-binding domain at the carboxy terminus, which is involved in the ubiquitylation and the elimination of misfolded proteins via the aggresome pathway [109].

The link between HDAC6 and HIF1A activity results from the regulation of HSP70/HSP90 chaperones [71,110]. HDAC6 can also deacetylate HIF1A and increase its levels and transcriptional activity under hypoxia. Curiously, both the deacetylase and ubiquitin-binding activity of HDAC6 contributed to the stabilization of HIF1A, but only deacetylase activity was required for the increase in HIF1A-mediated gene transcription [111].

## 8. Class III HDACs: Sirtuins

Sirtuins are a family of NAD^+^-dependent deacetylases that regulate several important cellular processes, responses, and fates. Sirtuins are present in different subcellular compartments, including the mitochondria. Their action is not limited to the removal of acetyl groups, but they can also control other PTMs such as succinylation or glutarylation [112]. Initial studies focused their attention on Sirt1, but it soon became clear that several Sirtuins can modulate the activities of HIF1A and EPAS1/HIF2A.

SIRT1 is redox-sensing and can stimulate EPAS1/HIF2A transcriptional activity during hypoxia. SIRT1 forms a complex with EPAS1/HIF2A and reverses lysine acetylation [113]. Conversely, SIRT1 can inhibit HIF1A by deacetylating K674, which is acetylated by PCAF. In this way, the binding of p300 and the transcriptional activity of HIF1A are reduced [114]. 

The results on SIRT1, HIF1A and hypoxia are contradictory, and show both neutral, positive and negative effects. The heterogeneity of these results is explained by the context-dependent effect of SIRT1 on additional targets [113,115,116,117]. Conflicting results have also been reported for the regulation of SIRT1 expression during hypoxia, with some studies indicating a suppression of SIRT1 transcription during hypoxia-induced epithelial–mesenchymal transition, or cancer stem cell-like properties [118,119,120]. This relationship is also confirmed during aging. Indeed, HIF1A is significantly higher in aged mice, while SIRT1 levels decrease. During hypoxia, SIRT1 was downregulated, allowing the acetylation and activation of HIF1A. Chronic activation of HIF1A promoted apoptosis and fibrosis [121]. In contrast, another study showed that SIRT1 expression is increased under hypoxia in a HIF1A-dependent manner. In a kind of positive feedback loop, SIRT1 was able to maintain HIF2A, but not HIF1A-mediated transcriptional activation of the isolated SIRT1 promoter [122]. Finally, DNA damage affects the activity of SIRT1 and the acetylation of H3K27 and HIF1A through the consumption of NAD^+^ [123]. In general, several reports indicate that a reduction in SIRT1 levels/activities correlates with increased acetylation of HIF1A and increased protein stability.

SIRT2 is a regulator of cellular metabolism and can influence the stability and transcription of HIF1A. Under hypoxia, SIRT2 deacetylates HIF1A at Lys709, but under normoxia, it can promote the instability of HIF1A [124]. SIRT2 plays an important role in the regulation of neuronal survival [125]. Similar to SIRT1, SIRT2 may also be part of a regulatory feedback loop regulated by HIF1A [126].

Mitochondrial and cytoplasmic SIRT3 could regulate HIF1A levels/stability in an indirect manner. SIRT3 monitors the level of ROS production, which may promote the stabilization of HIF1A [127,128]. The contribution of SIRT3 as a negative regulator of HIF1A stability has also been confirmed in other studies [129,130,131,132].

SIRT4 is another, mainly mitochondrial Sirtuin [133]. SIRT4 can negatively regulate aerobic glycolysis and suppresses HIF1A expression in pancreatic cancer [134]. This effect also appears to be mediated by ROS formation. However, in clear cell renal cell carcinoma, preliminary data suggest that SIRT4 interacts directly with HIF1A and may reduce HIF1A protein levels [135].

SIRT6 has attracted attention for its role in regulating of chromatin structure and DNA repair, as well as its involvement in aging and longevity [136]. Originally, SIRT6 was characterized as an epigenetic effector of HIF1A that acts as an H3K9 deacetylase. SIRT6 was required for the regulation of nutrient stress responses [137].

Overexpression of SIRT6 in human umbilical vein endothelial cells (HUVECs) can prevent HIF1A degradation by increasing deubiquitylation at K37 and K532. This action monitors the expression of HIF1A under both normoxia and hypoxia [138]. In another work, deacetylation of HIF1A in response to capsaicin treatment, driven by SIRT6, resulted in degradation of HIF1A [139]. 

SIRT7 has also been identified as a negative regulator of both HIF1A and HIF2A activity. The proposed mechanism is quite peculiar, and does not involve enzymatic activity, but a physical interaction [140]. This role was recently confirmed in vivo using *sirt7*-null zebrafish. Here, sirt7 facilitates polyubiquitylation and degradation of hif-1αa, hif-1αb, hif-2αa and hif-2αb, the two orthologous copies of *HIF1A* and *EPAS1/HIF2A* in zebrafish. These animals are more resistant to hypoxic conditions and are characterized by an increased expression of hypoxia-responsive genes and an increased number of erythrocytes compared to their wild-type counterparts [141].

## 9. Inhibitors

The involvement of HDACs in the regulation of hypoxia has been exploited therapeutically, not only in cancer, but also in several other diseases such as ischemia and pulmonary hypertension [142,143,144,145]. The effect of HDACIs on HIF1A activities and the hypoxia response were the initial evidence for the subsequent investigation of the contribution of various KATs and HDACs to HIF1A and EPAS1/HIF2A activities. The effects of HDACIs could be more indirect and explained at multiple levels. For example, as an adaptive response to treatment with HDACIs, cells reduce the expression of highly expressed genes [146].

HDACIs may also affect the stability of mutant pVHL, which is misfolded and unstable. By inhibiting the HDAC-HSP90 chaperone axis, HDACIs stabilize pVHL, restore its activity, and regulate HIF1A-dependent gene expression [147].

In soft tissue sarcoma (STS), SAHA/vorinostat can upregulate EPAS1/HIF2A expression but not HIF1A expression. HIF2A levels are downregulated in STS, and this downregulation supports cell proliferation and mTORC1 signalling. In contrast, its expression inhibits the growth of high-grade STS cells in vivo [148].

During hypoxia, translation of cap-dependent mRNAs is inhibited, while mRNAs encoding HIF1A and proangiogenic, hypoxia and survival factors undergo cap-independent enhanced translation [149]. Examination of changes in acetylome after treatment with the HDACI MS-275 revealed that Y-box-binding protein 1 (YB-1/YBX1) controls translation under stress conditions. YB-1 is an RNA-binding protein (RBP) that binds to the 5′- and 3′-untranslated regions (UTRs) of various mRNAs, including HIF1A [150]. Its binding to RNA is inhibited by lysine-81 acetylation. Therefore, HDACIs and acetylation may also indirectly control HIF1A levels. Indeed, a possible contribution of HDACIs to HIF1A translation through an undetermined action of HDAC9 was originally proposed [106].

These few examples illustrate the complexity of the effects that HDACIs may have on the response to hypoxia. Therefore, the discussion of HDACIs and hypoxia requires a detailed and comprehensive review that is beyond the scope of this manuscript.

## 10. Conclusions

HIF1A and HIF2A are two key players in the cellular adaptive response to reduction in O_2_ availability. O_2_ directly controls their activity by driving degradation via the UPS. However, HIF1A and HIF2A are subject to further controls to fine-tune their activities. These controls are mainly induced through PTMs, which rapidly adapt cellular activities to environmental changes. Here, we have discussed phosphorylation and acetylation of HIF-1 TFs as important additional options for modulating their transcriptional activities. In some cases, particularly with HDACs and the effects on the activities of HIF1A and HIF2A activities, the results are sometimes contradictory. The possible contribution of additional factors to the regulation of HDACs (including their epigenetic modulations) and the specific lysine residues involved need to be investigated. Only when this information is available will the contribution of HDACs to the activities of HIF1A and HIF2A be clarified. Further experimental work is certainly required to understand the fine regulation of the hypoxia response.
